# Midwives’ practice challenges in triaging expectant mothers: a qualitative study in Mpumalanga maternity units, South Africa

**DOI:** 10.3389/fmed.2025.1610724

**Published:** 2025-06-26

**Authors:** Mxolisi Welcome Ngwenya, Livhuwani Muthelo, Melitah Molatelo Rasweswe, Tebogo Maria Mothiba

**Affiliations:** ^1^Department of Nursing Science, University of Limpopo, Polokwane, South Africa; ^2^Faculty of Health Science, University of Limpopo, Polokwane, South Africa

**Keywords:** triaging, practice challenges, expectant mothers, maternity units, midwives

## Abstract

**Background:**

In the early decades, triage systems were universally implemented in accident and emergency departments. However, in recent years, various maternity triage systems, including digital technology triage models, have been gradually employed to evaluate the urgency of arriving pregnant women in maternity units. These systems face practice challenges, especially in South Africa, where data on triaging practices in maternity units are scarce, particularly in Mpumalanga province. Hence, this study seeks to identify and describe the practice challenges influencing the triaging of expectant mothers by midwives working in the maternity units of Mpumalanga province.

**Methods:**

A qualitative, exploratory descriptive design underpinned this study. The maximum variation purposive sampling technique was used to select midwives working in the maternity units of Mpumalanga province. Semi-structured interviews were conducted for data collection, and data saturation was reached and confirmed with the 20th participant. Thematic analysis was employed for data analysis.

**Results:**

The study highlighted that the triaging of expectant mothers by midwives is plagued by numerous practice challenges. Among these challenges were a shortage of resources, poor infrastructure, and incompetence among midwives. Nonetheless, the most concerning practice challenge was poor leadership, evidenced by the midwives’ viewpoints that there is a lack of support, whether informational (triage policies/protocols) or psychological.

**Conclusion:**

These findings suggest a need to rethink healthcare service delivery priorities to enable a clinical environment that improves the provision of quality care. Therefore, urgent action is required to develop context-specific triage protocols and guidelines for maternity units in Mpumalanga province. Moreover, multifaceted support programs aimed at empowering midwives and fostering a blame-free, non-toxic environment should be designed and implemented. Addressing the identified challenges will not only improve the provision of care but will also enhance the patient experience.

## 1 Introduction

In the early decades, triage systems were universally implemented in accident and emergency departments. However, various maternity triage systems, including digital technology triage models, are being gradually employed to evaluate the urgency of arriving pregnant women in maternity units, aiming to allocate the appropriate level of care in a timely manner (1). The maternity triage systems and tools implemented globally include the Obstetric Triage Acuity Scale (OTAS), Emergency Severity Index (ESI), Maternal Fetal Triage Index (MFTI), Birmingham Symptom-specific Obstetric Triage System (BSOTS), Florida Hospital Obstetric Triage Acuity tool, Self-Assessment Questionnaire for Gynaecological Emergencies (SAQ-GE), and Perinatal Emergency Team Response Assessment (PETRA) (2). Fakari et al. noted that these maternity triage tools are essential as they improve the quality of maternity care, reduce delays in accessing emergency care, and increase patient satisfaction with maternity care services (2). Similarly, Floyd et al. in Ghana concurred that the implementation of the triage system reduced third delays and optimized the quality of maternity care (3). McCarthy et al. agree with Floyd et al. that the introduction of maternity triage decision aids in maternity units not only enhances the quality of maternity care but also improves staff confidence, competency, and prioritization of care (3, 4).

Although the gradual implementation of triage systems is observed in several countries both within and outside Africa, some scholars have highlighted multiple practice challenges impacting the effectiveness of these systems (5). The Nova Scotia College of Nursing indicated that midwives often encounter professional practice challenges when providing midwifery care. These challenges can affect the quality of patient care and the midwives’ ability to deliver care consistent with their scope of practice and maternity care guidelines (6). Among these challenges are physical space, resistance, and midwives’ attitudes toward the triage system (5, 7).

However, in South Africa, data on practice challenges surrounding triaging in maternity units and the existence of a standardized triage system are scarce, particularly in Mpumalanga province. A recent study by Tukisi et al. in North West province reported that midwives felt their existing maternity triage tool was inadequate (8), leading to poor triaging of pregnant women. Moreover, midwives in the Tukisi study noted that ineffective triaging was exacerbated by issues such as poor midwife-to-patient ratios, high patient influx, and resource shortages (8). Several studies have highlighted that implementation challenges and practice difficulties of maternity triage are associated with poor maternal and perinatal outcomes. Oduro et al. affirmed that ineffective triaging contributes to maternal mortality due to the third delay. One study indicated that about 97% of preventable maternal mortalities are associated with this third delay (9). This issue was also noted in South Africa, where 450 maternal deaths were reported due to delays in accessing maternity care services (10). The Saving Mothers report indicated that out of the 450 maternal mortalities, 265 were due to an overburdened healthcare system, resulting in ineffective triage practices. Approximately 67.1 deaths per 100,000 live births were reported from Mpumalanga province in 2020. Several authors linked these deaths in Mpumalanga province to shortages of equipment and resources. Moreover, it is one of five South African provinces reported to have a high mortality rate related to preventable causes (10). Against this background, this study seeks to identify and describe the practice challenges influencing the triaging of expectant mothers by midwives working in maternity units in Mpumalanga province. This study is part of a larger project that highlighted the lack of standardized triage systems in maternity units of Mpumalanga province. In the absence of such a standard, the midwives indicated they had to use alternative triage approaches, which were impacted by various practice challenges. They often triaged patients based on professional instincts and experience, with some relying on patient clinical history and physical inspection as their primary triage techniques. These techniques were often influenced by consultations of guidelines and their clinical experience in emergency units. The details of these practices are outlined in another study published in a different journal (11).

## 2 Methods

### 2.1 Study design

A qualitative, exploratory, and descriptive design underpinned this study to discover knowledge about the practice challenges influencing the triaging of expectant mothers by midwives. This methodology assisted the authors in gaining a broader understanding and insights into the phenomenon of inquiry (12). This research approach allowed key participants to articulate and reflect on their shared theoretical understanding of triage and the practice challenges affecting effective triaging in their maternity units (13).

### 2.2 Study setting and sampling

**Figure 1:**
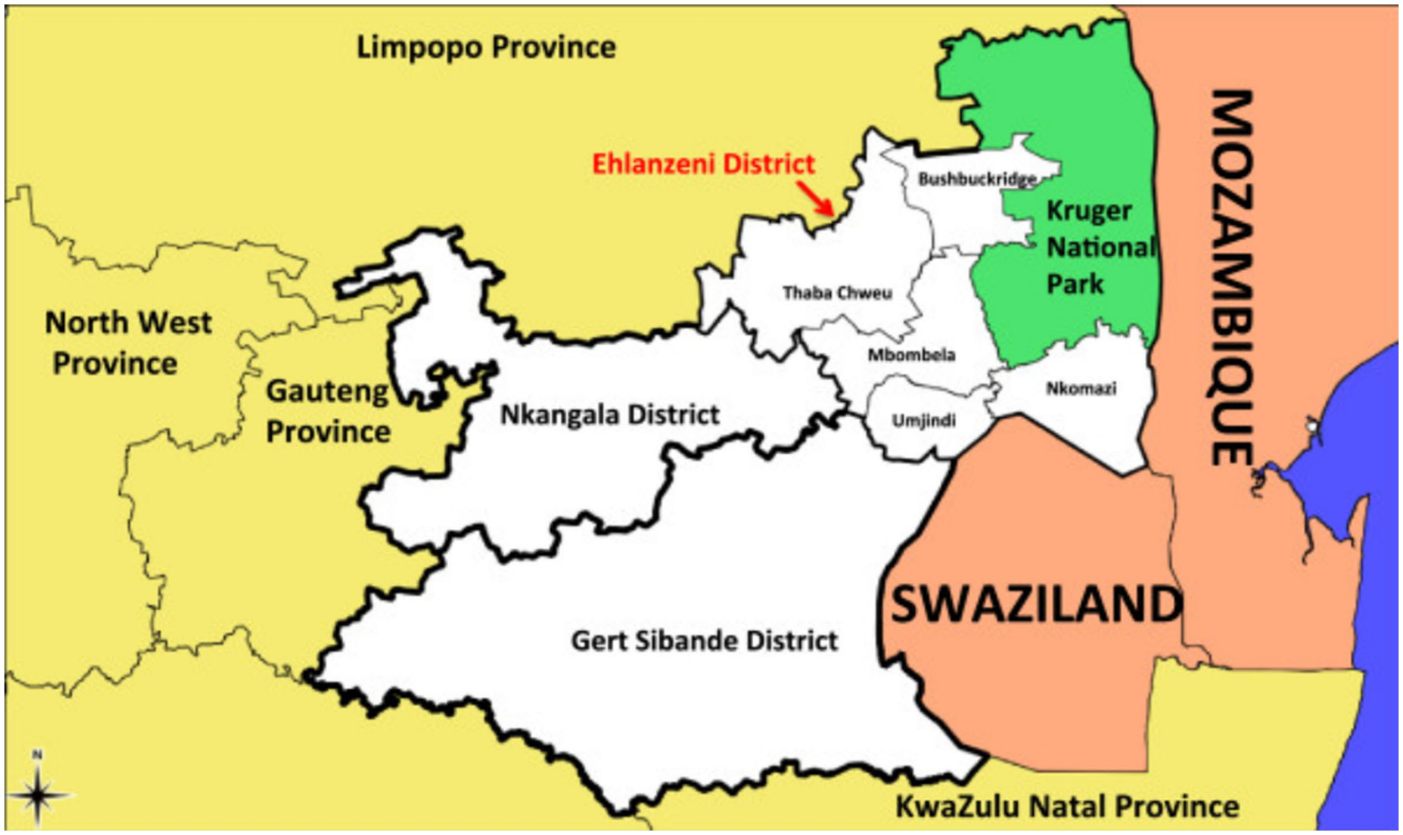
Map of Mpumalanga province showing bordering provinces and countries (34).

The study was conducted in district hospitals that were distinctly dispersed from each other, providing a broader representation of different districts within Mpumalanga province. This allowed the authors to gather a comprehensive view of the phenomenon of inquiry from the midwives. The hospitals provide comprehensive maternal health services to surrounding communities and receive referrals from local primary health care and community health care centres. The rationale for selecting district hospitals was highlighted by the recent Saving Mothers report, which indicated that over 60% of maternal deaths occurred in district hospitals, with one of the causes being the third delay (10). The selected hospitals were more than 295 km apart from each other, located in the Ehlanzeni and Nkangala districts of Mpumalanga province. As of 2022, the Ehlanzeni district had a population of about 2,270,897, while the Nkangala district had a population of 1,568,684. These two districts are at the receiving end of borders with Gauteng, Limpopo province, and international countries such as Mozambique and Swaziland (14). This is depicted in Figure 1; Mpumalanga province is shown in white in the figure. Nonetheless, maximum purposive sampling was adopted to select midwives working in the maternity units of the selected district hospitals. The midwives share characteristics, such as being registered midwives with more than 1 year of experience in providing midwifery care services, but they are from different district hospitals. This approach allowed the authors to gain rich insights into the phenomenon of inquiry from various perspectives (15).

### 2.3 Data collection and analysis

Data were collected through individual semi-structured interviews with the midwives. The semi-structured interviews followed a guide with a set of discussion questions to ensure that the research questions and objectives were addressed. The interview guide was self-developed following a literature search aligned with the research objectives and is attached as supplementary material 1 (interview guide) of this study. The interview process took place from May to July 2024 and was conducted by the first author in a private room during the midwives’ free time to minimize disruption to the provision of maternity care for the patients. These interviews were audio-taped and lasted for 30–50 min. The first author approached the data collection process with an open and objective viewpoint, remained neutral throughout the interviews, and kept his opinions to himself. Communication techniques such as probing, paraphrasing, clarification, reflection, and minimal verbal responses were used to gather rich, detailed data from the midwives. Data collection continued until saturation was reached, confirmed with the 20th participant. This was indicated by the lack of new insights and information arising from the final interviews with the 15th-20th participants. These last few interviews yielded no new information, thus confirming that data saturation had been achieved. The data were then transcribed verbatim and anonymized. Subsequently, the data were coded using thematic analysis. The analysis process began with familiarization with the transcripts, followed by identifying similar patterns in the dataset, which were interpreted into their inherent meanings. Potential themes were then developed and conceptualized from the identification of the codes and patterns that were grouped, thus forming themes. The analysis was conducted by all authors, who then met to reach consensus and refine the emerging themes.

Nonetheless, trustworthiness in this study was ensured through the Lincoln and Guba elements of trustworthiness. To enhance credibility, the authors employed peer debriefing by consulting with other scholars to obtain their insights on the findings before making inferences. Moreover, the authors engaged in rigorous triangulation through the utilization of more than one source of data collection. This involved triangulating the field notes with the interviews. Data from each facility were then triangulated to reach robust conclusions on the phenomenon of inquiry. About nine participants were interviewed in the first district hospital, followed by 11 participants from the second district hospital. Lastly, the authors attempted to ensure authenticity by faithfully reflecting the participants’ feelings and tones through the interpretation of the field notes.

## 3 Results

### 3.1 Participants’ demographic findings

A total of 20 participants were interviewed from two district hospitals in Mpumalanga province. The demographic findings revealed that the vast majority of the participants were female, with most holding a diploma in Nursing and a post-basic qualification in advanced midwifery. Additionally, approximately 11 of the participants were aged between 40 and 49, which aligns well with their level of experience, as more than 11 of the participants had over 10 years of work experience in midwifery. The demographic findings are depicted in Table 1.

**Table 1:** Midwives’ demographic data.

Age (years)	20–29	2	Highest qualification	Bachelor’s degree	4
	30–39	2		Diploma in nursing (R425)	7
	40–49	11		Advanced diploma in midwifery	2
	50–59	5		Post-basic qualification in advanced midwifery	7
Gender	Male	1	Work experience	1–5	6
				6–10	3
	Female	19		11–15	5
				More than 15	6

### 3.2 Main findings

The midwives in this study indicated that their triage practices are influenced by a variety of practice challenges. These challenges are institution-related, midwife-related, and patient-related. The subthemes of the practice challenges related to triage practices were supported by eleven categories. The themes and subthemes are reflected in Table 2.

**Table 2:** Themes related to practice challenges.

Themes	Subthemes
1. Institution-related practice challenges	1.1. Absence of support from management
	1.2. Equipment malfunctions, lack of supplies, and equipment are concerns
	1.3. Paradoxical explanations of protocols and policies
	1.4. Insufficient availability of human resources
	1.5. Poor infrastructure, inadequate physical space, and an inconducive environment related to overcapacity
	1.6. Difficulties in reaching other healthcare personnel
2. Patient-related practice challenges	2.1. Language barrier
	2.2. Lack of honesty from some of the patients
	2.3. Variations in lack of understanding and emotional upheaval versus understanding when redressed about triaging
3. Midwives’ related practice challenges	3.1. The ineptitude of midwives and poor teamwork skills in managing obstetric emergencies and conditions
	3.2. The existence of attitude and behaviour is a concern

### 3.3 Theme 1: institution-related practice challenges

The study indicated that the triage practices of the midwives are influenced by numerous practice challenges. Among these challenges, the midwives expressed concerns regarding unsupportive management, resource shortages, and inadequate infrastructure. The midwives further noted that the lack of policies and protocols, as well as difficulties in obtaining second opinions, added significant stress when triaging.

#### 3.3.1 Subtheme 1.1: absence of support from management

Most midwives expressed that they do not receive much support from management. Instead, they felt unsupported and isolated. Surprisingly, the role of support provided by management to employees was viewed as non-existent by the midwives. This was supported by the following narratives:

*“We do not get support, and they do not even bother…raising both hands with frustration,” P7.*

*“Our management does not support us with anything because even if you report that we have a challenge… I do not see anything that the management helps us with. Instead, we are getting strained, the eyes were wide open.” P1.*

Some midwives attributed the lack of support to the perception that regardless of the practice issues they reported, they were never acknowledged. They stated that the only time they interact with management is when an incident has occurred within the labor ward.

*“…because even if you report that we have a challenge, we do not have staff, and we are 2 in the ward, and the bench is full, we have many patients. They do nothing. You’ll work by the 2 of you as you are, and when problems come, they bring those papers of the Perinatal Problem Identification Program and tell you to fill in, saying that they heard that a certain patient gave birth on the bench and the baby died or stuff like that.” P1.*

*“No, we do not, because the matron (referring to the nursing service manager) rarely comes this side unless we have a big incident; she’ll come, but other than that, we do not get any support.” P4.*

It appears that the lack of support from management had a psychological impact on the midwives. One of the midwives associated this lack of support with feelings of demotivation. The participant further expressed that they only receive blame from management when a particular incident occurs rather than a pat on shoulder. The lack of interaction and support from management makes the midwives feel abandoned. It seems as though the midwives yearn for psychological support from their management. Another participant, looking dispirited and downhearted, said:

*“…they only come when we have had an incident to take the statement of what happened. But when you complain asking for enough equipment, enough staff. They do not respond to such things so that is the reason I’m saying we do not get support.” P7.*

#### 3.3.2 Subtheme 1.2: equipment malfunctions and lack of supplies are concerns

The study indicated that the lack of supplies and equipment was a pressing issue among the midwives, particularly when it came to patient care. This shortage negatively impacts the quality of care that patients are supposed to receive upon arrival at the maternity unit until discharge. Most of the midwives expressed their concerns about shortages and malfunctions of equipment. With a serious expression, the participants said:

*“Shortage of equipment, like currently we do not have urine strips. A woman comes in and reports signs of eclampsia, how are you going to be able to see how much protein is there in the urine without urine strips. So, we have a big challenge with the shortage of equipment” P18.*

*“We do not have enough equipment, like CTG machine. We only have 1 admission room, then sometimes you find that the BP machines are not working and the patient has been referred from clinic with a hypertension, so our BP machines does not want to record or do anything. Currently the urine dipsticks are out of stock also.” P5.*

*“We have many practice challenges. Equipment is a challenge as well. So, we have staff shortage and also the shortage of working equipment. We do not have enough equipment. the linen we do not have. Especially when we conducting deliveries. We have three delivery rooms but only two are working; the third one is there but we are not using it because the beds in there are not comfortable enough that you can conduct a delivery there. Isn’t there are different kinds of beds, these ones are the like the casualty ones, stretcher like…so we use two, but sometimes that many patients delivered at the same time, now that becomes a problem and now, we end conducting delivering into beds…. So, it makes the work difficult…” P19.*

Functioning equipment and sufficient supplies appear to be what the midwives need to provide quality care, particularly when triaging. The midwives strongly believe that to triage effectively, they require adequate equipment and resources. In some way, the study findings imply that for the midwives to meet the patients’ urgent needs, they need working equipment and resources. In addition, the lack of equipment such as cardiotocography (CTG) poses challenges for the midwives in providing care. The midwives emphasized that the lack and malfunctioning of equipment lead to delays in interventions and increased instances of stillbirths.

*“… Another reason for FSB is lack of equipment’s to place woman of continuous CTG monitoring for early identification of risks…” P14.*

*“…now, our level of care has dropped, as you can see, we are using one machine in this whole ward. The first CTG is now flat. When are you going to repeat that CTG. By the time you repeat that CTG, you find out that now there’s no foetal heart or there are complications…. But if we had enough equipment and staff, we would not even delay a patient.” P7.*

#### 3.3.3 Subtheme 1.3: paradoxical explanations of protocols and policies

The midwives indicated that another concern regarding triaging was that they lacked supporting protocols and policies. This meant they had no sense of legislative guidance on how to triage within the labor ward. The participants said,

*“We do not have protocols that support us with triaging, I’ve never seen any” P13.*

*“I do not remember seeing any protocols… The last time I saw triaging protocols it was the time I worked at casualty. But here I’ve never seen any protocols regarding triaging.” P5.*

Another participant elaborated further;

*No, we do not have protocols. We just work according to our experience.” P6.*

In the closing discussion, the findings indicate that labor wards lack protocols and policies to support triaging. This is evident from the contradictory extracts where midwives referenced the maternal guidelines for intrapartum management and induction. As a result of this lack of policies and protocols, the midwives stated that they rely on their experience to triage. There is seemingly a necessity for the establishment of triage protocols to support the midwives with triaging.

#### 3.3.4 Subtheme 1.4: insufficient availability of human resources

The midwives specified that the shortage of staff negatively impacts their triage practices. As a result, pregnant women tend to wait longer in the waiting area, and those waiting for review are also delayed. The midwives voiced that the shortage of staff contributes to poor monitoring of patients. It was noted that the poor triage practices among the midwives were due to being short-staffed, consequently causing imbalances in the patient-to-midwife ratio. Looking disappointed and unenthusiastic, the participants said;

*“I think it is difficult to triage. It is difficult in a way that when the patients are sitting here, we are unable to triage because we are short staffed. Everyone is busy with a patient, so when are you going to triage? At the end of the day, you find that a patient has already delivered while waiting on the bench whereas if we had enough staff, we’d be able to triage correctly.” P1.*

*“Usually because of the shortage, because we are short staffed here…” P4.*

Some midwives went on to elaborate on the ongoing staff shortages within the ward and said;

*“So, number one its staff, we have a shortage… The ward is allocated 4 midwives, and when you are 4 midwives and having 10 deliveries, C/section and 20 admissions it then becomes difficult for us.” P13.*

*“Shortage of staff. Because, as I’ve said, when they delegate, some you find out that they put midwives for admission. Like you said, you find that on the bench may be patients are six patients. Now, maybe when I check the first patient, I find out maybe full dilated or she’s having foetal distress. No I have to give her attention. And the other midwife takes another patient, now let us are three remaining in the waiting area. Not long after day one. Some of them, will end up delivering a baby there in the waiting area. Now we have to leave the patients we are currently attending to and run to attend to the one in the waiting area who just delivered. We have a shortage of staff… Like I said, staff is a challenge, now let us say am assisting a patient with a delivery and am alone, I have to take care of the baby and I have to take care of the mother. Sometimes, you find the woman with Postpartum haemorrhage, now I have to give oxytocin, and I also have to attend to the baby and try to prevent hypothermia. In some situations, it happens now two patients deliver at the same time and you are alone, it once happened to me. Now you have to change gloves and go to the patient, change again, and go back to the patient. You will end up mixing things, so you see, staff shortage is a problem…” P10.*

The midwives also noted that the shortage may be a result of the fact that whenever a midwife resigns or leaves the institution via transfer, they are never replaced, or it takes time to replace them. This leaves a significant gap in human resources that is not filled in a timely manner. At certain times, the posts for the maternity unit were advertised, but when those midwives are hired, they are often assigned to another ward, still leaving a human resource gap in labor wards.

*“When person leaves, let us say one nurse resigns or transfer. They hardly replace those nurses. As far as I know I do not how’s the recruitment process, we are not sure. But, when someone leave the hospital sometimes, they are not replaced. So, I’m not sure what is happening on that part.” P9.*

*“Because we also have a problem where posts are advertised for maternity professional nurses, but then at the end of the day when those nurses are hired you only find that they’ll bring only one or two nurses to the maternity but they said these hired professional nurses are for the maternity.” P4.*

Overwhelmingly, the issue of staff is concerning because the gap in human resources affects the triage practices of midwives. The midwives assert that in the labor ward, there should be a midwife specifically delegated to triaging patients upon arrival, rather than performing any other duties. It was suggested that this method would be very effective for triaging. In some sense, this will alleviate the pressure on staff; another participant mentioned that the waiting area is like another ward on its own. The midwives also stated that the shortage of staff impacts patient care even within the ward, as they sometimes end up having to multitask, such as conducting two vaginal deliveries alone. This might lead to mixing up babies, losing babies to hypothermia, and cross-infection among mothers and babies. Furthermore, the midwives mentioned that the shortage of staff negatively impacts their mental health and wellbeing. This was evident when the midwives expressed feelings of tiredness and burnout. Seemingly drained and dispirited, the midwives said;

*“…shortage of staff and overcrowded hospital leading to burnout…We are burnt out because of too much workload, overcrowded ward and shortage of staff” P14.*

*“…challenge is the tiredness, the fatigue from the staff.” P3.*

#### 3.3.5 Subtheme 1.5: poor infrastructure, inadequate physical space, and an unconducive environment related to overcapacity

The midwives strongly stressed that practice challenges such as poor infrastructure, lack of physical space, and an unconducive environment negatively influenced their triage practices. According to the midwives, the space was not big enough, and the unit was structured in a way that does not permit effective triaging. The participants strongly stated;

*“Our structure is small because the environment structure is not good…” P9.*

*“We do not have enough rooms for working, and we also do not have enough space for patients… And also, our working space like this one does not have basins. So, after helping the patient to deliver, there’s no place where you’d wash your hands around here it’s either you’ll wash them back at the admission room or elsewhere.” P17.*

Other midwives continued to elaborate on the challenges within the ward, noting that the hospital is quite small. However, this was attributed to overcrowding and the high number of patients within the ward. Most of the midwives said that because of the overcrowding, it makes their work difficult, and some patients end up sleeping on the floor. This was further emphasized in the following statements;

*“When we take them, we put them in the rooms because we do not have any special places like enough labour rooms, we only have 1 labour room… We do not have enough space, you’ll triage a patient and take her to where? We’re always full here, you find that mostly we have many c/sections. The problem is space. Our maternity ward does not have enough space. It is 3 in 1, and the next thing we are doing is nursing sick babies in this department. Or the one who’s just delivered there, if she’s not okay and you put her on oxygen, she’ll stay for some time there. They’ll tell you that the ICU beds are full, Paediatrics is full as well. So, you end up nursing her there and giving her treatments there, so at what time are you going to triage because you’ll be busy with the one who’s crashing there and needs to get treatment? So, it is difficult working in the maternity ward.” P1.*

*“We get more patients in this ward, and sometimes we do not know how to allocate them. Like now, where they are supposed to sleep, we are lacking beds. You find some others sleeping on the floor, others at the bench. So, we do not have enough space and rooms for working…” P17.*

*That’s the challenge that we have, that the hospital is small and we are between the 2 borders. But the bigger problem is Mozambique, they are the ones that make our labour ward overpopulated and we then fail to nurse them accordingly.” P13.*

*“I do not know if we’d be repeating the same thing. Our labour ward is overcrowded, we have many patients” P12.*

In summary, because of the lack of physical space, another midwife expressed concerns about confidentiality. When the rooms are full, pregnant women end up sharing beds, as the rooms cannot accommodate all the patients. The midwives emphasized the need for more space in the labor wards. One midwife mentioned that management had previously attempted to address the space issue; however, the problem persists. The midwives suggested that the overpopulation is also linked to the influx of migrating patients who come to deliver at the institution.

#### 3.3.6 Subtheme 1.6: difficulties in reaching other healthcare personnel

Within the realm of institution-related practice challenges, the midwives highlighted the concern of not being able to reach other healthcare workers. The difficulties in contacting other healthcare workers arose from the malfunctioning switchboard. Another midwife said, sometimes you triage, and then you want to reach out for a second opinion, but you find out the switchboard system is down. The midwives vividly described that sometimes the struggle is finding doctors during obstetric emergencies. This was supported by the following narratives:

*“What we do, we do triage and call doctors and senior staff in the labour ward …So, the thing of the switchboard sometimes does not work, we are not able to reach the doctors sometimes.” P16.*

*“…our phone is not working; you cannot reach the doctor. Like on weekends, like on Saturday, the phone was disconnected.” P3.*

*“Others would be that, sometimes, the doctor is not available at the time when he’s needed. Maybe we have a fitting patient of eclampsia and you have just taken her from the bench and put her on the bed. Then, when you call the doctor, you find that he’s not around. Now you have to start afresh and do everything and at that time the doctor is not around. So, the doctor sometimes is the one who gives us problems.” P1.*

### 3.4 Theme 2: patient-related practice challenges

Within the realm of practice issues, patient-related practice challenges were lucidly described by the midwives. For example, the midwives reported encountering challenges such as language barriers, particularly with patients from other African countries; lack of honesty from patients; and misunderstandings about triaging among patients.

#### 3.4.1 Subtheme 2.1: language barrier

The midwives highlighted the challenge of language barriers when triaging pregnant women. They noted that most patients they encountered with language issues were from outside South Africa. This was supported by the following quotes:

*“Like we have a language barrier from Mozambique patients. Some do not hear anything completely. They talk Portuguese, and none of us knows that here. That’s the problem we come across… And those who are not South African citizens, they give us many problems. You find that we are now at the delivery room and you cannot even tell them to push because they do not hear you at all.” P13.*

*“…some of them, especially since we are next to a foreign, we are next to Mozambique, so we receive a lot of foreign patients, so sometimes it’s a lot, sometimes we have language barrier issues” P11.*

The language inefficiencies between the patients and midwives interrupt the dynamics of healthcare. Consequently, the midwives were unable to gather important information related to clinical history and chief complaints upon arrival at the labor ward. One midwife expressed the necessity for a language translator within the labor ward, as this would enable them to obtain comprehensive patient histories through the translator.

*“…we are having difficulties with the, with those who have come from Mozambique and stuff, so they need like an interpreter, and also they need like files, you cannot just write when you have, you do not have enough details of the patient…” P11.*

#### 3.4.2 Subtheme 2.2: lack of honesty from some of the patients

The midwives expressed concerns about the lack of honesty among patients. With serious expressions, they stated that a lack of honesty impacts the triage process. Some midwives noted that some of the patients attributed the lack of honesty to be associated with the fear of being shouted at by midwives. This is supported by the following quotes:

*“… others go to the clinic and say they are on gravida 3, whereas they are on 6, so she’s hidden all the other 3. When she gets here at the hospital, we ask her what number this baby is, and she’ll say 6, and we say, but then on the file it’s written 3. And she’ll be at the clinic last time I told them I’m delivering the 5^th^ baby, they shouted at me so now I’m afraid to say this one is the 6^th^. So, when she gets to you, you have to know what gravidity she is because gravida 2 and gravida 6 cannot be treated the same. Gravida 6 needs you to know that this person is at high risk…” P13.*

*“Sometimes the patients do not tell you their full history, the truth that what’s going on. Let us just say she comes here because she drank something and that is why she’s having that precipitated labour, you see. Or else maybe, I do not know how to explain this… It is possible that a person kept time sitting with the problem, let us just say, like maybe she felt that the baby is no longer playing but she continued staying at home” P12.*

Other midwives highlighted another challenge is lack of honesty when it comes to booking for antenatal care. For example, there was one midwife who reported that the patients come to labor ward not booked for antenatal care and then lie that they did. Looking concerned, one midwife said that such situations complicate triaging. In contrast, another midwife mentioned that others lie and say they did not book for antenatal care only to find out they are indeed booked. The midwives assumed these patients were trying to hide the fact that they were on certain medications.

*“Like other patients, come here saying they are un-booked only to find out that they are booked. They are taking certain treatments and they hiding it from us” P7.*

#### 3.4.3 Subtheme 2.3: variations in lack of understanding and emotional upheaval versus understanding when redressed about triaging

In the context of patient-related practice challenges, midwives expressed varied experiences regarding patients’ understanding of triaging. Some midwives emphasized that some patients do not understand triaging in labor wards. They also noted that the lack of understanding of triaging among patients is often accompanied by emotional turmoil and upheaval. This was corroborated by the following narratives:

*“They do not understand it. They treat the labour ward like the clinic system, they will say that nurses are not attending to us. They will be complaining, saying that we take whoever, while she arrived first. They do not understand that we take a patient according to urgent needs first. Even after we explained it to them, we always explain it. But you can tell she is not happy about that at all.” P10.*

*“They do not feel okay, even though they might not say it, but then we know they spread rumours around the community saying that we attend our relatives first, we are unfair and stuff…” P18.*

*“…And others shout and fight that they came in first, you see things like that, so it becomes difficult to triage.” P1.*

*“So, they think we do not care about them. Because they always expect to take the one that arrived first, and they feel like they are not well taken care of… So yeah…” P9.*

The midwives further noted that some patients do not understand the triage process even after it has been explained to them. One midwife stated that this makes triaging very problematic. Another midwife said,

*“…so, we explain to them that an emergency came in, so we will start with it, so the patient who has been waiting for some time gets angry and finds it unfair to them, and they do not understand.” P7.*

With a serious face, another midwife downheartedly said:

*“…we try to be patient with them, but they do not want to be patient with us…” P11.*

On the other hand, other midwives argued that the lack of understanding among patients is due to a lack of explanation during triaging. Patients tend to understand and agree with the midwife. This was supported by the following quote:

*“The patients need you to explain to them first because you cannot just show up and choose. So, you have to explain to them that “I know you came in first, but this one need urgent assistance, so can I please take her in first?” P1.*

*“It’s like if you find them sitting, when you check and see that there’s one that is ready to deliver and you take her in, others will understand…” P13.*

In the closing discussion, most of the midwives consensually said that it differs with patients when it comes to triaging in the labor ward. Some midwives elaborated that even when explained in detail, some patients still respond with, *“I am in pain, cannot you see…” P9.*

### 3.5 Theme 3: midwives-related practice challenges

Despite the patient-related practice issues, the midwives expressed that they experience other practice challenges. Among these challenges, the midwives outlined their concerns regarding the skills and competence of midwives in managing obstetric emergencies and foetal wellbeing via CTG monitoring.

#### 3.5.1 Subtheme 3.1: the ineptitude of midwives and poor teamwork skills in managing obstetric emergencies and conditions

The midwives highlighted that they often experience a lack of teamwork and collaboration skills among team members. They strongly emphasized that some of the causes of ineptitude and poor teamwork skills are related to laziness. In conjunction with a voice of concern, the midwives said;

*“I think the challenge from the ward is a shortage only, and on top of that, we have those who do not want to work. Like, sometimes a patient needs assistance at the bed, and the other nurses ignore them, and she just disappears.” P7.*

*“Ahh, I think that would be laziness. Maybe other staff would be lazy to do this, I think…” P6.*

On the other hand, the midwives further elaborated on their concern about the lack of skills among other midwives, which is accompanied by a lack of motivation to fulfil their duties. As a result, some midwives do not intervene in certain obstetric problems when the need arises. Instead, they try by all means to pass the duties to other midwives. This was corroborated by the following assertions;

*“There are people who are still behind, but they do not care when working. Yeah, they do not take things seriously, or the problem seriously. They just shift the problem to another person, saying that the next person will see on their own, which is a problem…The results are bad because you are working because you are in a hurry to knock off, not because you want to help the patient, which is wrong. Or you leave a problem, then say the other person will see it, which is wrong” P9.*

*“Some lack skills, even when they see there’s a problem, they do not act. Some do not know how to perform CTG.” P5.*

#### 3.5.2 Subtheme 3.2: the existence of attitude and behaviour is a concern

In the process of triaging, the midwives highlighted that another major factor is behavioral, such as the attitude of the midwives. One midwife expressed that there is an attitude among the midwives when providing care. This was corroborated by the subsequent assertions.

*“Mostly, patients are complaining about the attitude of nurses. So, I think if we are to change our attitude first and see things on the same level, we’d go far. And also focus on working together.” P5.*

Individually, the midwives assumed that the existence of attitude among them is rooted in several factors. Some attributed the attitude to the tiredness of midwives; meanwhile, others said that the attitude emerges when midwives are asked questions. These were supported by the following narratives;

*“…challenge is the tiredness…And the attitude, because we are now tired. The attitude we give each other.” P3.*

*“…others they do not like to be asked questions, or she gives you an attitude, you see…” P10.*

## 4 Discussion

A study by Rominski et al. briefly highlighted that triage is a procedure focused on prioritizing patients based on their level of emergency while ensuring that they receive time-appropriate care (16). This contemporary study reports on practice challenges that influence effective triaging of expectant mothers by midwives in maternity units in South Africa. These practice challenges ranged from institutional-related, midwives, and patient-related issues. The study deepens our understanding of why third-delay maternal and perinatal mortality and morbidity are still national crises in South Africa. Most importantly, the study opened our eyes to crucial gaps that need strengthening in the healthcare system to improve effective triaging.

Most of the midwives in this study reported facing difficulties during the triage process. The findings identified a lack of support, shortage of equipment, inadequate infrastructure, staff shortages, and lack of protocols or policies as the prominent challenges in triaging in maternity units. Similarly, the studies of Moudi et al. (7) and Rashidi-Fakari et al. (5) reported that lack of human resources, unavailable protocols, and poor infrastructure negatively influence the triage practices of midwives. These challenges were also seen in studies conducted in the emergency departments of other hospitals. For instance, a study by Bijani et al. reported that an inconducive work environment with a shortage of resources and a lack of clear triage protocols influences the quality of triage (17). Thus, influencing the midwives’ decision-making when triaging. The lack of protocols and clear guidelines was also indicated by the studies of Tukisi et al. (8) and Reynolds et al. (18). Those studies highlighted that a lack of policies and protocols contributes to ineffective triaging among midwives, putting the lives of pregnant women and their babies at risk. This suggests an essential call to policymakers to develop policies and protocols aimed at improving triage practices of midwives in labor wards. The use of standard operating policies, such as standardized assessment tools, provides guidance and appropriate methods to assess pregnant women facing clinical challenges. Thus, policies and protocols related to maternity units can improve the reliability of care and avoid taking the incorrect clinical pathway (19).

Nonetheless, in juxtaposition with the findings to the WHO framework of quality maternal and child healthcare, it was no surprise to learn from the study findings that such specific concerns within the healthcare system still exist in the South African context. Although the WHO framework emphasizes that human and physical resources are essential for improving and optimizing quality maternal and child healthcare (20), some studies in earlier decades concurred with the study findings that the shortage of resources and malfunctioning equipment are major concerns (21). However, despite the economic status of SA, the healthcare system is still a work in progress. This was indicated in the 2nd presidential health impact report 2024–2029, which highlighted nine pillars to achieve a quality and reformed healthcare system. Among the nine pillars are the expansion of human resources, execution of the infrastructure plan, and development of an information system to guide policies and protocols (22).

Despite the burning desire to strengthen the healthcare system to optimize effective triaging in maternity units, the findings identified language barriers and a lack of understanding of triage among patients as critical practice challenges. This seemingly harms the midwife–patient communication channel. Similarly, a study by Wittenberg et al. (23) highlighted that a lack of understanding impacts midwife–patient communications, particularly when patients have low health literacy levels. It is often this lack of understanding that affects the provision of care. Shaban et al. underscore that understanding plays a key role in patient care, and the moment a patient is understood, the real healing process begins (24). The WHO quality framework concedes that effective communication with patients and their families is crucial as it responds to their needs and preferences. It is an essential component of the care experience that can significantly reduce anxiety during childbirth and create a positive experience for women (20, 24). Therefore, this study underscores the dire need for midwives to return to the foundations and core values of nursing, where building trusting relationships and health education were cornerstones of healthcare services. Furthermore, this study recommends that midwives provide health education concerning triage, focusing on its importance and relevance in maternity care to cultivate patients’ understanding of its necessity. In addition, midwives reported that language barriers are a significant concern, often hindering their ability to obtain consent and deliver healthcare services. Several scholars also indicated that a lack of understanding related to language barriers is linked to insufficient information, often associated with delayed diagnosis, impacting the patient experience of care (25). However, communication challenges, such as language barriers, affect not only patients but also midwives. A study conducted in South Africa reported that these challenges create work burdens and frustrations among midwives, leading to feelings of being overwhelmed (26).

On the other hand, the findings of this study highlighted a lack of honesty among patients. Midwives noted that pregnant women often do not disclose their health status and current complaints honestly when presenting to labor wards, typically coming to the hospital without a history of attending prenatal care (un-booked). One midwife expressed that some patients lie about their HIV status and parity. This lack of honesty may indicate a lack of trust and fear. There is limited literature on why pregnant women do not fully disclose during triage; however, Mule et al. argue that the lack of honesty among pregnant women is rooted in self-judgment, fear of being judged by midwives, the value of privacy, and a lack of trust in healthcare personnel. Nevertheless, the reasons for not fully disclosing information differ based on the patient’s condition (27). Thus, this study recommends future research avenues to explore patients’ perspectives on not fully disclosing their health status during triage.

The study identified that midwives also face practice challenges such as a lack of urgency, poor teamwork, and insufficient support. These concerns relate to the lack of proficient skills among midwives. The Saving Mothers Report concedes that these issues may be associated with avoidable maternal deaths (10). For instance, the study by Moodley et al. indicated that problems like these are linked to delays in recognizing emergencies, contributing to increased rates of maternal and perinatal mortality and morbidity (28). The Saving Mothers 2020–2022 Triennial Report on Confidential Enquiries into Maternal Deaths in South Africa indicated a total of 1,032 cases of suboptimal care with probable poor outcomes and maternal deaths, with about 450 of these cases attributed to the third delay. These cases indicate poor maternal quality of care (10). To address this, Harris et al. indicated that establishing effective teamwork skills is crucial for patient-centred quality maternity care (29). Downe et al. reiterated that creating a collaborative culture is essential, particularly in maternity units where some cases require emergency care and treatment. Interventions in maternity units necessitate teamwork and collaboration to improve patient outcomes (30). According to Wrammert et al., working in a team introduces diverse members with varied competencies and experiences, and this collaborative practice ensures positive outcomes for patients (31).

On the other hand, midwives expressed that the complexities of maternity units, such as a lack of support and resources, often leave them feeling burned out and tired. The midwives said that instead of support, they receive blame from the senior management. They never received a pat on the shoulder for good work. According to the UK Parliament Committee, the culture of blame in maternity units is often associated with perinatal and maternal mortality. The committee further highlighted that this culture prevents lessons from being learned and creates a dysfunctional team and defensive culture (32). In this contemporary study, midwives indicated that senior managers often visit the maternity unit only after poor maternal and perinatal outcomes, providing no support. Some scholars propose strategies that emphasize the importance of a blame-free culture while promoting positive attitudes and quality improvement (33). It is suggested that maternity units foster a collaborative and supportive environment, as findings in this study indicate a strong desire for psychological support among midwives. Moreover, this calls for stakeholders to develop guidelines and interventions that promote collaborative practices and support among midwives and senior management. Drawing from the overall findings, it appears that strengthening the healthcare system to achieve effective triaging and meet Sustainable Development Goal 3 targets depends on the availability of resources, protocols, and an enabling clinical environment. Considering the healthcare system building blocks proposed by the WHO, components such as leadership and governance, human resources for healthcare, medical products, and quality health service delivery are essential for quality improvement in this study.

## 5 Limitations of the study

The study has contextual limitations, as it was conducted only in selected district hospitals in the Mpumalanga province. Therefore, the findings do not reflect a wider context, such as other regions or healthcare settings. This indicates a need for research studies examining broader contexts to reach robust conclusions regarding the phenomenon in question. The study illuminated practice challenges related to the triaging of expectant mothers presenting to maternity units in Mpumalanga province. However, despite identified theoretical similarities across the literature, the findings should not be generalized to other contexts or clinical departments.

## 6 Conclusion

The study underscored that the triaging of expectant mothers by midwives is plagued by numerous practice challenges, including resource shortages, poor infrastructure, and incompetence among midwives. However, the most concerning issues were poor leadership and a lack of understanding from patients and family members. This is evident in the midwives’ perspectives, which reveal an absence of support, whether informational (triage policies/protocols) or psychological. In addition, the study findings show variations in patients’ understanding of triaging. This variability is reasonable, as midwives shared diverse experiences with different patients. These findings suggest a need to rethink healthcare service delivery priorities to enable a clinical environment that improves the quality of care provision. Therefore, there is an urgent need to develop context-specific triage protocols and guidelines for maternity units in Mpumalanga province. Moreover, multifaceted support programs aimed at empowering midwives and fostering a blame-free, non-toxic environment should be designed and implemented. Addressing the identified challenges will not only improve care provision but also enhance the patient experience.

## Data Availability

The raw data supporting the conclusions of this article will be made available by the authors, without undue reservation.
